# Starch Sodium Octenylsuccinate as a New Type of Stabilizer in the Synthesis of Catalytically Active Gold Nanostructures

**DOI:** 10.3390/ijms25105116

**Published:** 2024-05-08

**Authors:** Beata Tim, Emilia Konował, Anna Modrzejewska-Sikorska

**Affiliations:** 1 Faculty of Materials Engineering and Technical Physics, Poznan University of Technology, Piotrowo 3, 60-965 Poznan, Poland; beata.tim@doctorate.put.poznan.pl; 2 Faculty of Chemical Technology, Poznan University of Technology, Berdychowo 4, 60-965 Poznan, Poland

**Keywords:** gold nanoparticles, sodium starch octenylsuccinate, catalytic activity, signal amplifiers

## Abstract

Here, starch derivatives, i.e., sodium starch octenylsuccinate (OSA starch, hereinafter referred to as OSA), were employed as both reducing and stabilizing agents for the unique, inexpensive, and simple synthesis of gold nanoparticles (OSA-AuNPs) in an aqueous solution with gold salt. The obtained OSA-AuNPs were characterized by UV-vis spectrophotometry, transmission electron microscopy, scanning electron microscopy, and energy-dispersive X-ray spectroscopy. The catalytic activity of the obtained gold colloids was studied in the reduction of organic dyes, including methylene blue (C.I. Basic Blue 9) and rhodamine B (C.I. Basic Violet 10), and food coloring, including tartrazine (E102) and azorubine (E122), by sodium borohydride. Moreover, OSA-AuNPs were utilized as signal amplifiers in surface-enhanced Raman spectroscopy. The obtained results confirmed that gold nanoparticles can be used as effective catalysts in reduction reactions of selected organic dyes, as well as signal enhancers in the SERS technique.

## 1. Introduction

The basic group of gold nanoparticles (AuNPs) are spherical nanoparticles; however, there are also rod-shaped, star-shaped, prism-shaped, and cube-shaped nanoparticles (NPs). Among the metallic NPs, AuNPs are distinguished by their unique catalytic properties [1,2,3]. In addition, the phenomenon of localized surface plasmon resonance occurring in NPs allows for the utilization of AuNP assemblies as enhancers in surface-enhanced Raman spectroscopy (SERS) and as photothermal agents [4,5,6,7]. 

The synthesis of metal NPs using green chemistry methods has the following advantages:

- Low process costs;
- Environmentally friendly processes;
- Ease of process scale-up;
- Prevention of pollution and waste generation;
- Energy efficiency;
- Economic viability [8].

In accordance with the requirements of the principles of “green chemistry”, environmentally friendly solvents are used for the synthesis of AuNPs, as well as ecological reducing and stabilizing agents, which are usually extracts of plant active substances, fungi, bacteria, lignosulfonates, and polysaccharides [9,10,11,12,13]. Among the first plant extracts used in the biosynthesis of AuNPs, extracts from the leaves of scented geranium (*Pelargonium graveolens*) [14], lemongrass (*Cymbopogon flexuosus*) [15], and tamarind (*Tamarindus indica*) [16] were employed. Over the years, NPs have been commonly prepared from other plant extracts, including common aloe (*Aloe barbadensis*) [17], magnolia (*Magnolia kobus*) [18], green tea (*Camellia sinensis*) [19], flowers of the aster family (*Achillea wilhelmsii*) [20], Japanese raisin tree (*Hovenia dulcis*) [21], edible hibiscus (*Abelmoschus esculentus*) [22], marine algae (*Galaxaura elongata*) [23], *Cochlospermum gossypium* tree [24], Indian almond (*Terminalia catappa*) [25], and onion (*Allium cepa*) [26]. 

In the synthesis of AuNPs, other stabilizing substances have also been used, such as fungi (e.g., Alternaria sp., Rhizopus oryzae, Aspergillus oryzae, Colletotrichum sp., Penicillium breyicompactum, Aspergillus Niger, Phanerochaete chrysosporium) [10,27], bacteria (e.g., Bacillus licheniformis) [28], carbon nanotube hybrids [29], lignosulfonates [30], peptides [31], enzymes [32], and polysaccharides (e.g., starch [33], chitosan [34]). 

Starch, which is a glucose polymer, is a commonly used polysaccharide for the synthesis of AuNPs. A characteristic feature of its structure is the linkage of monosaccharides into chains through glycosidic bonds. Starch plays a crucial role in the functioning of living organisms, serving as a storage and structural material. Examples of polysaccharides include starch, glycogen, cellulose, and chitin. Among the main characteristics of starch are renewability, non-toxicity, biodegradability, and easy availability. Depending on the botanical origin and variety, starch differs by fat content, protein content, amylose and amylopectin levels, degree of polymerization, and water-binding capacity. Wheat starch, for instance, has a higher fat content than corn or potato starch [35,36]. 

Modification of starch can improve its functionality, which includes esterification, polymer grafting, etherification, hydrolysis, and hydrothermal treatment [37]. Furthermore, modified starches play a significant role in the synthesis of NPs. Pienpinijtham and co-workers [38] presented the green synthesis of AuNPs using soluble starch as a reducing and stabilizing agent. Since soluble starch is a weak reducing agent, an alkalizing agent, sodium hydroxide (NaOH), was added to promote its decomposition and enhance these properties. During the reaction, a darkening of the yellow solution was observed, indicating the formation of NPs with spherical shapes. Zeng and co-workers [39] prepared AuNPs using starch from edible cassava (*Manihot esculenta*). The plant used in the study had a high polysaccharide content, which, upon liquefaction, released oligosaccharides, including reducing sugar. The reaction promoted the reduction of gold ions, resulting in a color change from pale yellow to red. The obtained NPs were mostly spherical, with sizes ranging from 15 to 35 nm and an average size of 22.8 ± 8.9 nm. Khan and co-workers [40] synthesized AuNPs using an extract from *Cotoneaster horizontalis*, which is mainly composed of starch, fatty acids, reducing sugar, and reduced Au^3+^ ions. The novelty of this method lies in its short synthesis time (approx. 5 min for AuNPs). The obtained NPs exhibited spherical shapes, with an average diameter of 18 nm, good stability, and a crystalline structure. Wongmanee and co-workers [41] presented the synthesis of AuNPs stabilized by starch biopolymer derived from mung beans, with trisodium citrate as the reducing agent. The observed color change during the reaction from light yellow to red indicated the reduction of tetrachloroaurate(III) anions. It was determined that the obtained AuNPs had spherical shapes, with an average size of 10 ± 2.5 nm. In the studies conducted by Castillo-López and co-workers [42], the biosynthesis of AuNPs using potato extract was presented, serving as both a stabilizer and a reducing agent. Potato starch, a component of the extract, participated in the reduction reaction of Au^3+^ ions and stabilization of the resulting NPs. They found that the obtained AuNPs had spherical shapes, with a size range of 17.5–23.5 nm. The main factor determining the stability of the colloidal solution was the pH of the reaction mixture. For NPs prepared in an acidic environment, stability was maintained for approx. 2.5 months. Meanwhile, NPs synthesized in an alkaline environment remained stable even after a year. Das and co-workers [43] presented the synthesis of AuNPs using a biodegradable copolymer composed of hydroxyethyl starch (HES), a derivative of amylopectin, and methyl acrylate (MA). The reaction required NaOH to achieve a slightly alkaline pH of 8, where a color change from yellow to violet occurred. The obtained NPs exhibited spherical shapes, with an average size ranging from 16 to 20 nm. Malathi and co-workers [44] synthesized AuNPs using isonicotinic acid hydrazide (INH) in the presence of starch as a blocking agent in an aqueous environment. The formation of the NPs was indicated by a color change of the solution to purple. The resulting NPs had monodispersity, with a spherical shape and an average size of 14.4 ± 2.7 nm. 

In the research section of this study, six types of OSA-modified starch were used for the production of AuNPs. OSA-modified starch, known as sodium starch octenylsuccinate, displayed a good ability to reduce interfacial tension in contrast to native starch, which does not exhibit such properties due to its hydrophilic nature [45]. One of the methods used for starch modification to obtain OSA-type starch is the esterification of octenylsuccinic anhydride in an alkaline environment. The alkalinity aims to reduce hydrogen bonding between starch chains. After modification, OSA-type starch becomes an effective emulsifier and stabilizer owing to the attachment of bifunctional groups, which are both hydrophilic and hydrophobic, resulting in its amphiphilic character [46,47,48].

NPs synthesized using green chemistry methods have found applications in various fields due to their environmentally friendly properties. AuNPs are of particular interest due to their unique characteristics, such as non-toxicity, biocompatibility, bio-stability, antibacterial properties, electrical and thermal conductivity, good surface activity, and optical properties. These features enable their use in electronics, optics, medicine, cosmetic industry, food industry, and catalysis [23,39,49,50]. Rapid advancements in AuNPs synthesis have led to a search for products applicable to the field of biomedical sciences [50,51,52,53]. AuNPs strongly absorb surface plasmons, leading to the generation of scattered light and heat, allowing them to be applied for sample labeling during transmission electron microscopy (TEM) and X-ray imaging. Moreover, they have been employed for diagnostic purposes and in photothermal therapy, leveraging their characteristics in photochemical treatments [54,55]. Nanomaterials are also of interest in electrochemical fields. AuNPs are used in devices such as biosensors, which are used in medicine, such as in the treatment of Parkinson’s disease, and in the detection of pathogens, viruses, or bacteria. They are also used for chemical analysis, for example, in the detection of metal ions [56]. It is known that the complex obtained from AuNPs stabilized with lignosulfonate and mercury ions can be used as a modifier for the electrode in the development of amperometric sensors. The resulting compound can then be applied for the detection of heavy metal ions, such as thallium [29].

The application of AuNPs in heavy metal sensors can be used to detect the presence of specific elements in water samples [57]. Another application of AuNPs is catalysis. Silver–gold alloys have been employed as catalysts for the reduction of 4-nitrophenol and picric acid in the presence of sodium borohydride for the synthesis of 4-aminophenol. AuNPs prepared from mung beans stabilized with starch (MBS-AuNPs) have also been used for the reduction of 4-nitrophenol [44,58]. 

In this paper, we synthesized AuNPs using modified starch of the OSA type. To confirm the formation of these NPs, UV-vis, TEM, and SEM analyses were performed, allowing for the estimation of the particle size. Additionally, the catalytic activity of the systems in the catalytic decomposition of dyes using sodium borohydride was tested, as well as their effectiveness as enhancers in the SERS (Surface Enhanced Raman Spectroscopy) technique.

## 2. Results and Discussion

**Figure 1:**
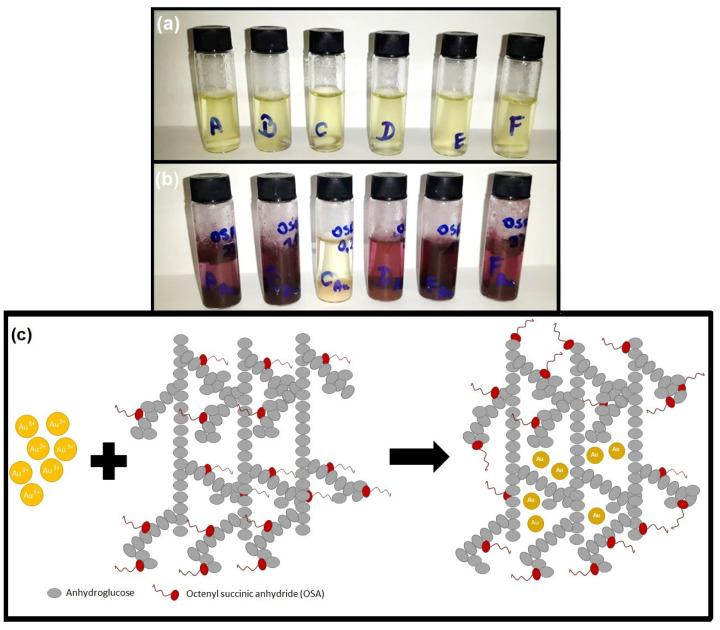
OSA-AuNP colloids: (**a**) 5 min after adding the gold precursor solution to the starch solution; (**b**) 24 h after adding the gold precursor solution to the starch solution. From left to right, solutions containing OSA starch: 2.0%, 1.5%, 0.2%, 0.5%, 1.0%, 3.0%; (**c**) schematic illustration of the fabrication of NPs.

The addition of the gold precursor solution to the starch solution in most of the studied colloids caused the mixture’s color to change gradually from light yellow to maroon (Figure 1a,b). The exception was the solution containing OSA 0.2, where no color change was observed. The schematic reduction process of gold ions to gold metal nanoparticles with OSA is represented in Figure 1c.

**Figure 2:**
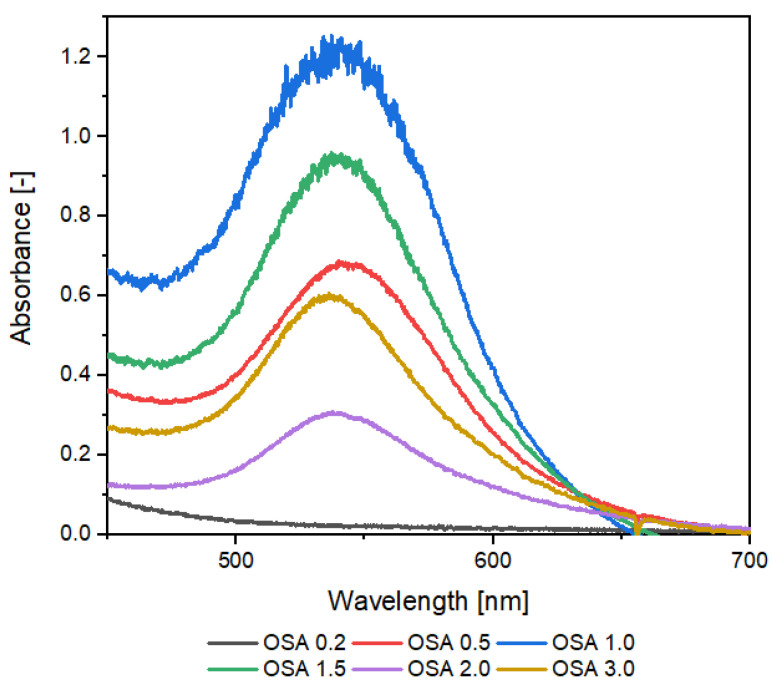
Absorption spectra of OSA−AuNPs.

The next step was to examine the optical properties of OSA-AuNPs using UV-vis spectroscopy. For selected OSA-AuNPs, the size and shape were examined using TEM. Also, SEM/EDS analysis was performed. As expected, UV-vis spectra (Figure 2) for structures stabilized with OSA starch above 0.2% showed an absorption peak at ca. 550 nm, which is characteristic of AuNPs. AuNPs stabilized with both OSA 3.0 and OSA 2.0 starch had a maximum absorbance at 536 nm. For AuNPs prepared with OSA 0.5, the maximum absorbance was 542 nm. We found that the concentration of AuNPs in the colloid stabilized similarly with OSA 0.5 and OSA 3.0. Analysis of the spectra showed that AuNPs containing OSA 1.5 and OSA 1.0 had the highest absorbance peaks at 539 nm and 537 nm, respectively. Therefore, these solutions had the highest concentration of AuNPs, which was evidenced by the intense color of the colloids (Figure 1). For AuNPs stabilized with OSA 2.0, the peak absorbance value was the lowest, which indicated the lowest concentration of AuNPs in the colloid. In the case of the colloid containing OSA 0.2, the color suggested the absence of AuNPs, and no absorbance peak was observed at approx. 550 nm.

The OSA substituent itself, from a chemical point of view, does not exhibit reducing properties and therefore does not play a direct role as a reductant of gold ions. Neither native starch nor OSA-modified starch usually have reducing properties. Starch consists of long chains of anhydroglucose units linked by α-1-4 glycosidic bonds (amylose) and α-1-6 glycosidic bonds at the branching points (amylopectin). This results in the appearance of a certain number of reducing ends, which depends on the type of starch and the degree of modification.

It is worth noting that OSA-modified starch shows surface activity [59,60]. Modification with OSA groups likely affects the spatial organization of starch chains, facilitating the reduction reaction of Au^3+^ ions to gold nanoparticles (AuNPs). The changes in spatial organization appear to be related to the content of OSA groups in the starch macromolecule. However, the process itself requires further, more detailed understanding, which will be the subject of further research.

**Figure 3:**
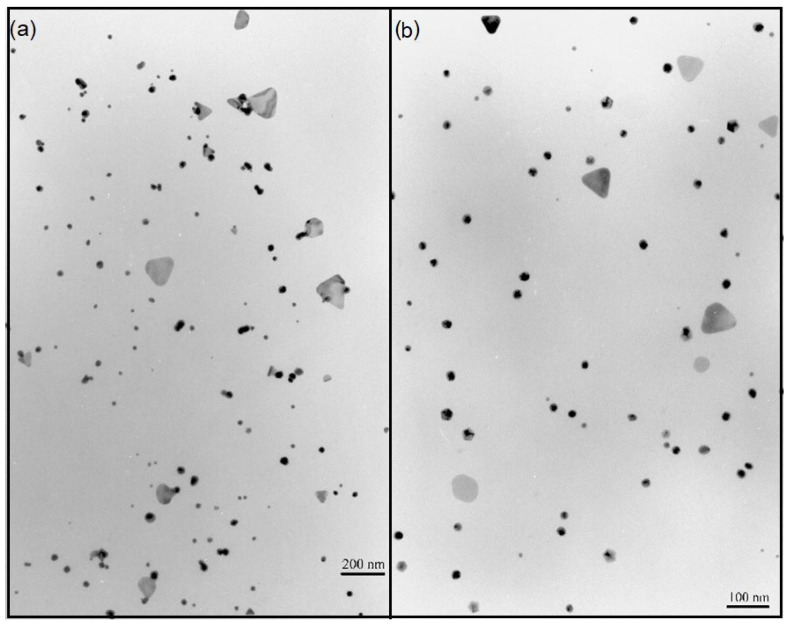
TEM images of OSA−AuNPs: (**a**) OSA 3.0, (**b**) OSA 2.5.

The microscopic analysis of TEM images of AuNPs stabilized with OSA 2.5 (Figure 3b) showed that the created NPs were primarily spherical in shape, and their average size range was 20–30 nm. Slightly larger triangular structures were detected, with an average size range of 50–60 nm. Also, NPs stabilized with OSA 3.0 (Figure 3a) assumed mostly spherical shapes, with an average size range of 30–40 nm. Also, there were triangular NPs present, with an average size of approx. 100 nm.

A comparative analysis of the results of the particle size distribution and absorption spectra shows that the larger plasmonic structures of OSA3.0−AuNPs (50–60 nm) have longer/wider absorption bands than the smaller (20–30 nm) nanoparticles of OSA2.5−AuNPs.

**Figure 4:**
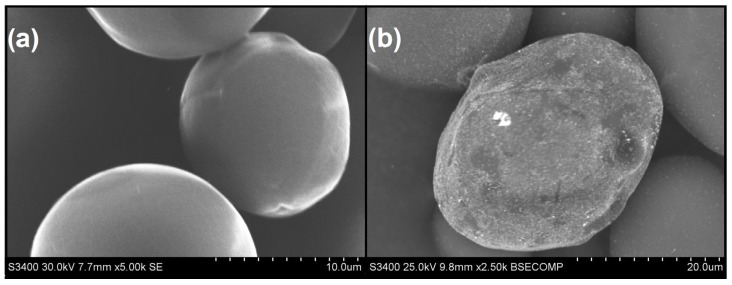
SEM images of OSA 3.0 starch grains: (**a**) before modification with gold precursor solution, (**b**) after modification with gold precursor solution.

Also, the microscopic analysis of SEM images (Figure 4) confirmed the formation of spherical AuNPs with OSA 3.0. Visible differences between the structure of OSA 3.0 before and after modification with a gold precursor solution verified the correct process of OSA-AuNPs synthesis.

**Figure 5:**
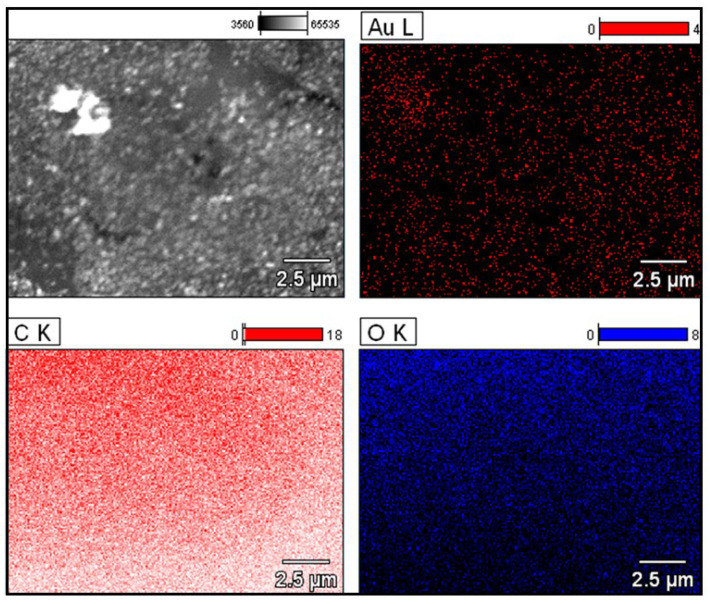
EDS analysis of OSA 3.0 stabilized nanostructures.

EDS analysis (Figure 5) showed the presence of gold in the structures stabilized by OSA 3.0, which contributed to their elemental composition determination. OSA-AuNPs were characterized by the presence of C, O, and Au, and their amounts are presented in Table 1.

**Figure 6:**
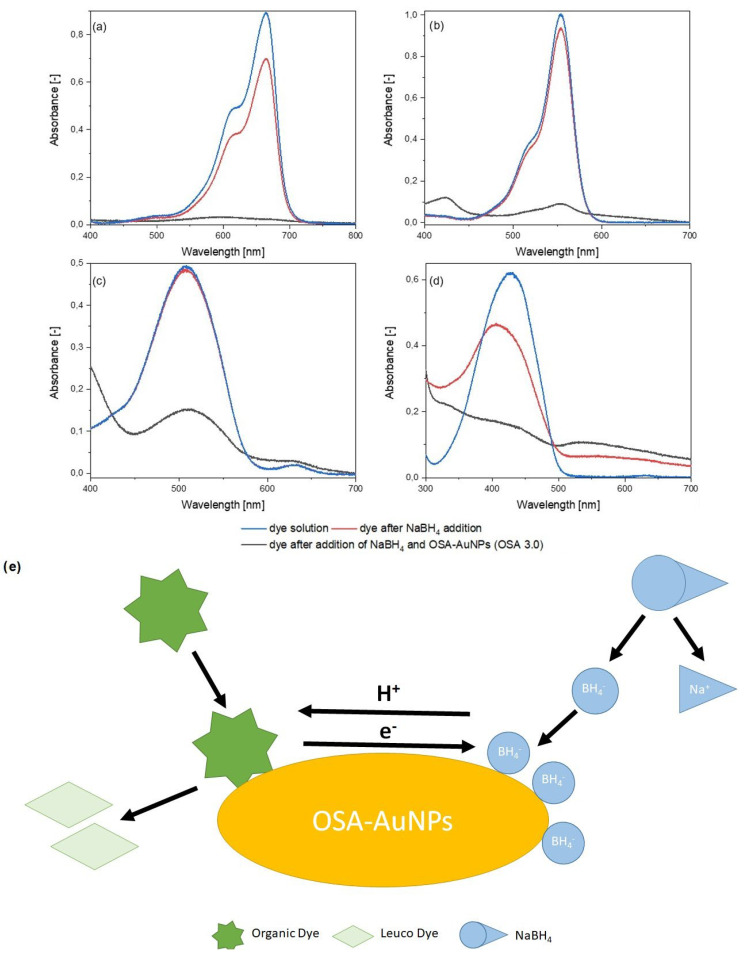
Absorption spectra of catalytic decomposition: methylene blue (**a**), rhodamine B (**b**), azorubine (**c**), and tartrazine (**d**); and the schematic illustration of the dye decomposition process using OSA−AuNPs (**e**).

AuNPs preparation via green chemistry methodology is widely described in the literature for applications in numerous fields. Therefore, the formed nanostructures must have the appropriate shape and size, but also the appropriate properties relevant to a particular application. Thus, the catalytic activity of OSA-AuNPs (OSA 2.5 and OSA 3.0) in the catalytic decomposition reactions of dyes to their leuco-form using one of the strongest and most effective reducing agents, sodium borohydride (NaBH_4_), was investigated. In chemistry, many reduction reactions are kinetically hindered, necessitating prolonged reaction times, which in turn may necessitate an excess of NaBH_4_ due to its low stability and auto-decomposition in aqueous environments. Incorporating gold or other nanostructures into the process could resolve the issue [12,61]. The decomposition of the studied substances could be ascertained by visual observation of the solution’s color change, which occurred due to the addition of the reducing agent and OSA-AuNPs. The discoloration of the solution indicated that dye decomposition occurred. Additionally, this was confirmed by UV-vis analysis (Figure 6). The kinetics of the substance decomposition reaction were investigated by observing changes in the intensity of the peaks occurring at the respective wavelengths. Spectral analysis revealed that, for each dye, the use of NaBH_4_ alone as a reducing agent did not cause significant decomposition of the test substance, and resulted in a slight decrease in the absorbance spectra. However, the addition of OSA-AuNPs accelerated the reaction, reducing the absorbance significantly. The rate of the decomposition reaction was different for each dye. This was mainly due to the differences in the chemical structure between individual dyes and various concentrations of the tested substances. In the case of methylene blue, the kinetics of the decomposition reaction were conducted by observing the peak intensity changes at 665 nm. For the reaction catalyzed by AuNPs stabilized with OSA 3.0 (Figure 6a), the complete disappearance of the maximum occurred 10 s after the addition of the nanostructures. For rhodamine B (Figure 6b), the peak at 555 nm was used to study the kinetics of the reaction. A reduction in the absorbance maximum from 1.00 to 0.15 occurred 10 min after the introduction of OSA-AuNPs into the system. For azorubine (Figure 6c), changes were monitored at a wavelength of 560 nm. The addition of OSA-AuNPs reduced the absorbance of the dye by 65% after 60 min. For tartrazine (Figure 6d), the peak at 430 nm was studied, where a significant decrease in absorbance occurred 60 min after the addition of OSA-AuNPs. In summary, the fabricated metal nanostructures were quite good catalysts for the decomposition of organic dyes. The schematic illustration of the decomposition process of dye using OSA-AuNPs is shown in Figure 6e.

**Figure 7:**
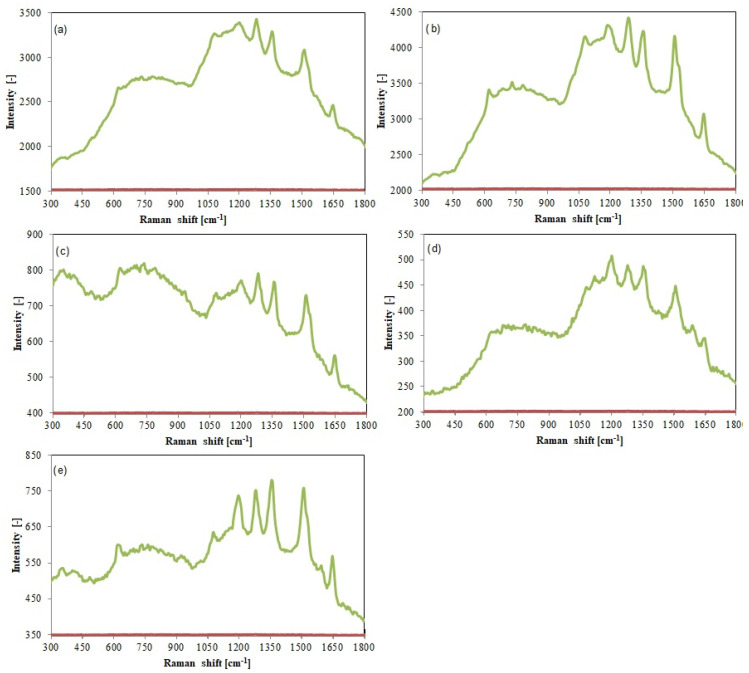
Comparison of Raman spectra of rhodamine B recorded on flat Au (red line) and OSA−AuNPs substrate (green line): (**a**) OSA 0.5, (**b**) OSA 1.0, (**c**) OSA 1.5, (**d**) OSA 2.0, (**e**) OSA 3.0.

Recent studies have shown that, in the field of amplification of the observed spectral signals in Raman spectroscopy, noble metal NPs have unique properties. For the rhodamine B solution, which was placed on an OSA-AuNPs layer or, for comparison purposes, on a bare Si/Au substrate, the Raman spectra were recorded. The obtained results (Figure 7) demonstrated that the rhodamine B spectrum recorded in the absence of AuNP nanostructures was featureless, whereas that recorded on a silicon wafer covered with colloidal gold showed the presence of peaks reflecting the intense vibrations of the bonds present in rhodamine B for nanostructures with a different degree of substitution with functional groups.

## 3. Materials and Methods

The study used sodium starch octenylsuccinate (OSA starch) with a degree of substitution, with functional groups of: 0.2% (OSA 0.2), 0.5% (OSA 0.5), 1.0% (OSA 1.0), 1.5% (OSA 1.5), 2.0% (OSA 2.0), 2.5% (OSA 2.5), and 3.0% (OSA 3.0) (Department of Food Concentrates and Starch Products of State Research Institute, Poznan, Poland). The methodology for the starch modification process and the method for determining the degree of substitution were described in [59,60].

Potassium tetrachloroaurate (III), sodium borohydride, methylene blue, and rhodamine B were purchased from Merck (Darmstadt, Germany). Also, food coloring—tartrazine and zzorubine (Pol-Aura, Morag, Polska)—was used.

The synthesis of OSA-AuNPs was carried out as follows. First, aqueous starch solutions (0.05 g·L^−1^) were prepared by dissolving the appropriate amount of solid OSA in de-ionized water at 50 °C (solution I). Then, an aqueous solution of KAuCl_4_ (1 g·L^−1^) in relation to Au^3+^ ions (solution II) was prepared. The prepared solutions were mixed 1:1 (*v*/*v*) and stirred for 24 h at a temperature of 50 °C. Both the colloidal solution obtained (UV-vis, SERS, TEM) and the resulting precipitate dried at room temperature (SEM, EDS, catalysis) were further analyzed.

Spectrophotometric analysis was performed with an Ocean Optics (Orlando, FL, USA) UV-vis spectrophotometer model USB4000. Structural analysis was performed using a JEOL (Tokyo, Japan) JEM-2200FS microscope. SEM and EDS analyses were performed using a Hitachi (Tokyo, Japan) microscope S-3400N equipped with an UltraDry EDS detector (Thermo Scientific, Waltham, MA, USA). The dried and powdered OSA-modified AuNPs starch was affixed to the microscope stage using the bulk method with a graphite disc. For SEM imaging, a thin layer of carbon (approximately 10 nm in thickness) was deposited onto the samples using a vacuum sputtering machine. SERS measurements were performed on a QE-Pro Raman spectrophotometer (Ocean Optics, Orlando, FL, USA) equipped with a diode laser (785 nm wavelength). Sample preparation for SERS measurements is described in detail in ref. [12]. The surface of the gold-coated silicon wafer was further modified with a gold colloid. After evaporation of the solvent, a thin layer of nano-gold remained adhered to the substrate surface. Then, a solution containing rhodamine 6G was applied to both the OSA-AuNPs layer and the bare Si/Au substrate, acting as a control, to evaluate the suitability of colloidal gold stabilized with starch OSA as a signal enhancer for the SERS technique.

The catalytic activity of OSA-AuNPs was measured based on the analysis of the optical absorption spectra. First, the spectrum of the aqueous dye solution (2 mL) was collected. Then, an appropriate volume of sodium borohydride (Merck, Darmstadt, Germany) (NaBH_4_, 5 g·L^−1^) was added to the test solution, and the spectrum was recorded. Finally, 0.05 g of solid OSA-AuNPs was added, and the absorbance was measured.

## 4. Conclusions

OSA starch, as an Au^3+^ reducer and stabilizer of the production of OSA-AuNPs, possesses gold nanostructures and catalytic properties. The results of our research highlight the possibility of a relatively simple production of large amounts of highly concentrated OSA-AuNPs with the use of inexpensive and readily available materials. The obtained OSA-AuNPs can be used as effective catalysts in reduction reactions of selected dyes to their leuco-forms with the use of NaBH_4_, as well as signal enhancers in the SERS technique.

## Figures and Tables

**Table 1:** Elemental analysis of OSA 3.0 stabilized nanostructures.

**Element**	**% wt**	**% at**
C	55.62	63.89
O	41.65	35.91
Au	2.73	0.19
Total	100.00	100.00

## Data Availability

Data are obtainable from the corresponding authors upon reasonable request.
